# Natural Polymorphisms Conferring Resistance to HCV Protease and Polymerase Inhibitors in Treatment-Naïve HIV/HCV Co-Infected Patients in China

**DOI:** 10.1371/journal.pone.0157438

**Published:** 2016-06-24

**Authors:** Kali Zhou, Zhiwei Liang, Charles Wang, Fengyu Hu, Chuanyi Ning, Yun Lan, Xiaoping Tang, Joseph D. Tucker, Weiping Cai

**Affiliations:** 1 Guangzhou Eighth People’s Hospital, 627 Dongfeng Dong Road, Guangzhou, Guangdong 510060, China; 2 University of California San Francisco, Department of Medicine, Division of Gastroenterology, 513 Parnassus Avenue, Room S-357, San Francisco, California, 94143-0538 United States of America; 3 UNC-Project – China, Division of Infectious Diseases, Department of Medicine, UNC Chapel Hill School of Medicine, 130 Mason Farm Rd., 2nd Floor, University of North Carolina Chapel Hill, Chapel Hill, North Carolina, 27599-3368, United States of America; 4 Brown University School of Medicine, Department of Medicine, Division of Gastroenterology 593 Eddy Street, APC 406, Providence, Rhode Island, 02903, United States of America; Centro de Biología Molecular Severo Ochoa (CSIC-UAM), SPAIN

## Abstract

**Background:**

The advent of direct-acting agents (DAAs) has improved treatment of HCV in HIV co-infection, but may be limited by primary drug resistance. This study reports the prevalence of natural polymorphisms conferring resistance to NS3/4A protease inhibitors and NS5B polymerase inhibitors in treatment-naïve HIV/HCV co-infected individuals in China.

**Methods:**

Population based NS3/4A sequencing was completed for 778 treatment-naïve HIV/HCV co-infected patients from twelve provinces. NS3 sequences were amplified by nested PCR using in-house primers for genotypes 1–6. NS5B sequencing was completed for genotyping in 350 sequences. Resistance-associated variants (RAVs) were identified in positions associated with HCV resistance.

**Results:**

Overall, 72.8% (566/778) of all HCV sequences had at least one RAV associated with HCV NS3/4A protease inhibitor resistance. Variants were found in 3.6% (7/193) of genotype 1, 100% (23/23) of genotype 2, 100% (237/237) of genotype 3 and 92% (299/325) of genotype 6 sequences. The Q80K variant was present in 98.4% of genotype 6a sequences. High-level RAVs were rare, occurring in only 0.8% of patients. 93% (64/69) patients with genotype 1b also carried the C316N variant associated with NS5B low-level resistance.

**Conclusions:**

The low frequency of high-level RAVs associated with primary HCV DAA resistance among all genotypes in HIV/HCV co-infected patients is encouraging. Further phenotypic studies and clinical research are needed.

## Introduction

The emergence of novel therapeutics for chronic hepatitis C virus (HCV) infection has brought this global pandemic to the forefront of public health attention [1]. Co-infection with HIV is common in HCV patients due to shared routes of transmission. One of the largest populations with HIV/HCV co-infection worldwide can be found in China, with a high proportion residing in the southern region as a result of intravenous drug use and drug trafficking from the Golden Triangle [2]. The long latency period from asymptomatic infection to cirrhosis and hepatocellular carcinoma with chronic HCV infection contributes to low uptake of HCV therapy in HIV co-infection [3], particularly when weighing the competing comorbidities of opportunistic infections and the high burden of pegylated interferon and ribavirin therapy. The introduction of highly active antiretroviral therapy normalized HIV life expectancy over time, thereby unmasking the morbidity and mortality of co-infection with HCV. End stage liver disease is now one of the leading causes of death in HIV-infected individuals [4]. Successful HCV treatment appears to mitigate this effect by slowing or stopping progression of fibrosis [5]. The advent of direct-acting agents (DAA) has allowed earlier and better tolerated treatment of HCV in HIV co-infection, increasing feasibility of HCV treatment uptake.

Several DAAs are currently approved for HCV treatment in the United States while many others are still in phase II and III trials [6]. DAAs are categorized as NS3/4A protease inhibitors, NS5B polymerase inhibitors and NS5A protein inhibitors depending on the viral protein that is targeted. Currently approved protease inhibitor therapies include telaprevir, boceprevir, and simeprevir in combination with peg-interferon and ribavirin for genotype 1 and paritaprevir in an interferon-free regimen. The NS5B polymerase inhibitor sofosbuvir is also available in an all-oral, interferon-free regimen and studies have demonstrated the feasibility of this regimen in HIV/HCV co-infection [7]. A second NS5B polymerase inhibitor dasabuvir is approved in a regimen including ombitasvir, paritaprevir and ritonavir. Currently treatment with DAAs is not available in China and many challenges in traditional HCV therapy remain including cost, low awareness of treatment options, low treatment uptake, and poor adherence [8]. An additional barrier is the diverse distribution of HCV genotypes among co-infected patients in China, the most common being genotypes 1b and 6a [2, 9]. Genotype 6 patients, infrequently seen outside of Southeast Asia, are seldom included in clinical trials, and treatment data are incomplete [10].

One key limitation of DAA treatment has been the presence of primary drug resistance leading to treatment failure. The highly error prone RNA polymerase of the hepatitis C virus accounts for the occurrence of HCV as an assembly of quasispecies in the human host, in which a low proportion of less fit variants with natural resistance-conferring polymorphisms can exist [11]. Treatment with DAAs provides selective pressure for these variants, particularly with protease inhibitors, which as a drug class has a lower threshold for developing resistance [12]. Virologic failure manifesting in 1–13% of patients enrolled in early clinical trials was nearly always associated with the detection of mutant variants at the time of breakthrough and a number of these variants were present prior to initiation of treatment [13]. While wild-type virus eventually repopulates the HCV population [14], the uncertainty of this timing and the potential for cross-resistance are current barriers to re-treatment. Presence of pre-existing resistance-associated variants (RAVs) such as R155K that lead to complete treatment non-response has also been a cause of concern, although rarely detected [15]. At this time, the consequences of DAA resistance, particularly prior to treatment, are not yet fully appreciated.

Studies investigating the occurrence of baseline DAA resistance are limited in Asia and most of the focus thus far globally has been on genotype 1. The aim of this study is to determine the prevalence of natural polymorphisms conferring resistance to NS3/4A protease inhibitors and NS5B polymerase inhibitors in treatment-naïve HIV/HCV co-infected patients in China across a broad spectrum of HCV genotypes.

## Material and Methods

### Patient selection

Plasma samples for NS3/4A sequencing were obtained from 826 HIV/HCV co-infected patients recruited between 2004 and 2013 from Beijing, Chongqing, Fujian, Guangdong, Guangxi, Heilongjiang, Henan, Hunan, Shaanxi, Xinjiang, Yunnan, and Zhejiang provinces. All patients had never received antiretroviral treatment or any HCV treatment. Demographic information was obtained at patient enrollment and extracted through chart review. Patients provided signed written informed consent prior to study participation. IRB approval was granted by the Guangdong Provincial Skin Diseases and STI Control Center Ethical Review Committee project number 57832455.

### HCV genotyping

**Fig 1:**
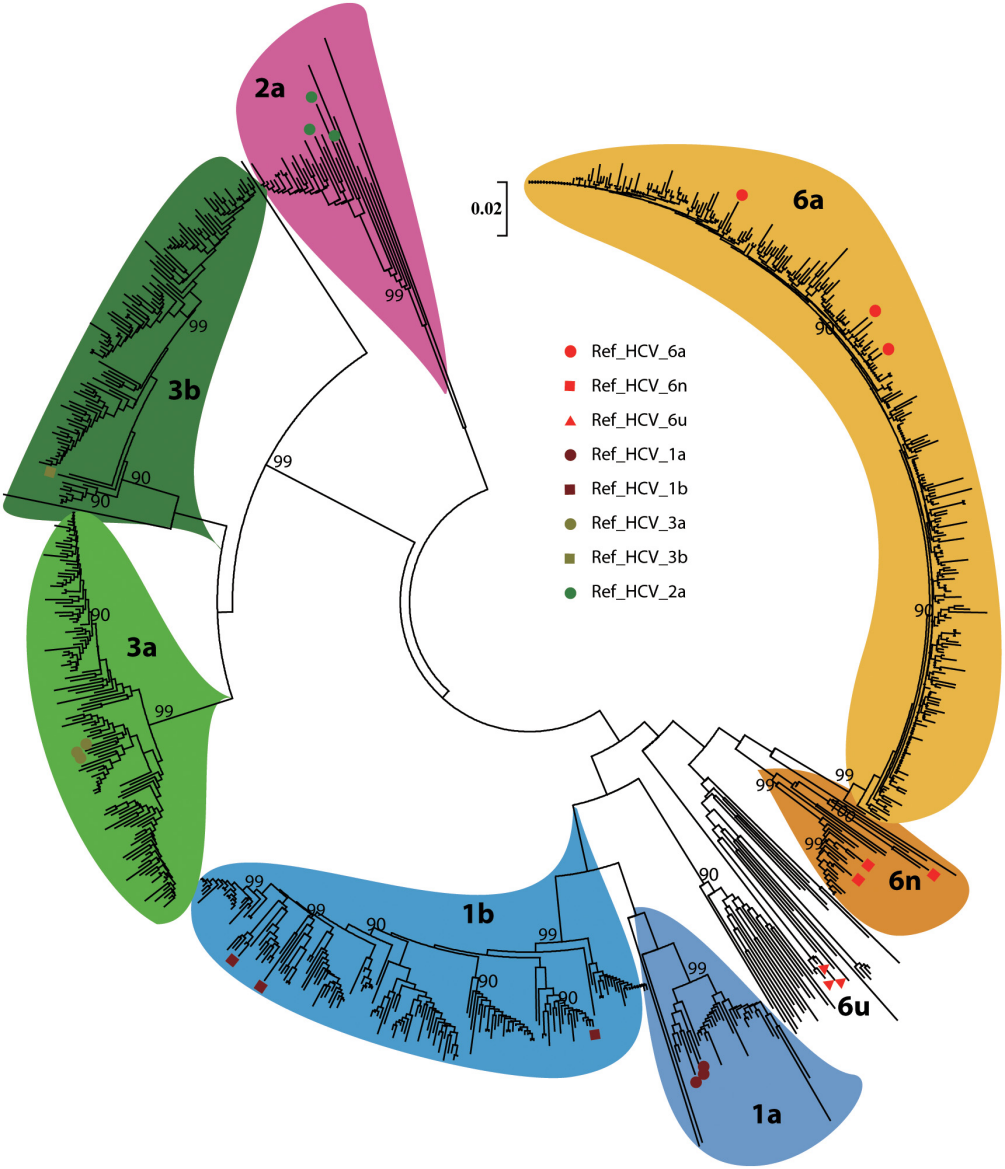
Phylogenetic tree. Neighbor-joining tree representing the relationship of all sequences with reference sequences. Solid circle (●) marks different subtype identified reference. Bootstrap analysis was performed with 1000 replicates and only bootstrap values ≥70% are shown at the corresponding nodes. Reference sequences: Genotype 1a (AF511950, NC004102, M67463); 1b (AY587016, D11355, EF032892); 2a (AY746460, D00944, AB047639); 3a (NC009824, AF046866, X76918); 3b (D49374); 6a (DQ480513, AY859526, Y12083); 6u (EU408332, EU408331, EU408330); 6n (EU246937, EU246938, AY878652).

HCV genotype and subtype were determined by RT-PCR and sequencing of the HCV core region. A subset of patients was sequenced using both HCV core and NS5B regions. Consensus sequences of the forward and reverse strands were used. Sequences obtained were compared against the Los Alamos HCV database [16]; reference sequences can be found in S1 Table. Genotype and subtype were then confirmed by phylogenetic tree reconstruction (see Fig 1) in MEGA v5.05 [17] using the neighbor joining method with the Kimura-2 parameter model. A gamma parameter of 0.5 was used to model differences in substitution rates among base sites. Branch support was assessed by bootstrap analysis with 1000 replicates.

### Sequencing of NS3/4A protease region

HCV RNA was extracted from serum with Viral RNA Mini Kit (Qiagen, USA), collected from the spin columns, and eluted with 60μl AVE buffer to obtain 55μl of virus RNA. Complementary DNA was then amplified based on viral template using M-MLV First-Strand Kit RT-PCR (Invitrogen, China). Because HCV has a very high frequency of variation in the region of NS3 gene, we designed genotype-specific primers to amplify the part of HCV NS3 containing potential DAA resistant mutations (primers are available in S2 Table). Primers were employed in nested PCR (Ex Taq DNA Polymerase, Takara, Japan) to target the NS3/4A protease-coding region. Sequences were obtained as a consensus of the forward and reverse strands. The conditions for the first round of nested PCR include: an initial denaturing step at 94°C for 4 minutes, followed by 30 cycles of a denaturing step at 94°C for 10 seconds, an annealing step at 55°C for 30 seconds, an extension step at 72°C for 2 minutes, and then followed by a final extension at 72°C for 10 minutes. Products of first round PCR then underwent second round processing consisting of the steps of 1^st^ PCR but with an annealing temperature decrease to 50°C. The final PCR product was detected by agarose gel (1.5%) electrophoresis and sent to sequence directly by ABI 3730XL automated sequencer using BigDye terminators (Applied Biosystems, Foster City, California, USA) by Invitrogen^™^ CO., Ltd (Guangzhou, China). A proportion of samples could not be sequenced due to quality of the sample and low viral load.

### Analysis of resistance-associated variants

NS3 sequences were aligned using BioEdit v7.0 [18]. Sequences for genotype 1a (Genbank accession number: AF009606), 1b (AJ238799) and 2a (AF177036) were aligned with ClustalW [19] according to reference sequences recommended by HCV-DrAG [20]. All other genotypes were aligned to the genotype 1a H77 reference sequence due to lack of established reference sequences. Insertions and deletions present were manually validated. The accession numbers for sequences submitted to Genbank are KT162143-KT162915. Variants were manually identified at positions reported to confer resistance to HCV protease inhibitors: 36, 41, 43, 54, 55, 80, 109, 155, 156, 168 and 170 [12, 21]. Association with phenotypic resistance and fold-change resistance data to approved DAAs and those in clinical trials were obtained from the literature [21, 22]. While there is no established cut-off between high-level and low-level RAVs, we used an EC50 > 10 to define a high-level RAV consistent with previously published literature [12]. In patients with NS5B sequencing, four positions reported to confer resistance to NS5B polymerase inhibitors could be identified in the region sequenced for genotyping (nucleotides 7935–8505): L159, S282, C316, and V321. Variants were similarly manually identified in these sequences.

### Molecular modeling

Crystal structure of simeprevir bound to HCV protease was obtained from the Protein Data Bank (PDB code 3KEE) in Swiss-model platform [23]. DeepView v4.1 was used for analysis of the Q80K variant and construction of a three-dimensional structure [24].

## Results

### Patient characteristics

The breakdown of genotypes in our study included 204 (25%) with genotype 1a/b, 25 (3%) with genotype 2a, 253 (31%) with genotype 3a/b and 344 (42%) with genotype 6a/n/u/v (see Table 1). Men comprised roughly two-thirds of the total population (76%). The majority of patients were young, between 30 and 40, with only 5% of patients older than 50 years of age. Intravenous drug use was the primary identified risk factor for transmission in 69% of patients and represented the majority of patients in genotypes 1, 3 and 6. Genotype 2 patients differed from other genotypes in blood transfusions as the dominant route of transmission, older age and a higher proportion of women. Clinical characteristics among the genotypes were similar with an overall median CD4 count of 126, median ALT of 38 units/liter (normal 7–56), median AST of 35 units/liter (normal 10–40), and log10 HCV RNA median of 6.1 copies/uL. The low median CD4 count reflects a high level of pre-antiretroviral therapy immunosuppression in our cohort.

**Table 1:** Demographic and clinical characteristics of all patients separated by genotype.

Characteristic	Total n = 826	Genotype 1 n = 204	Genotype 2 n = 25	Genotype 3 n = 253	Genotype 6 n = 344
Subtype					
a		33 (16%)	25 (100%)	124 (49%)	321 (93%)
b		171 (84%)	--	129 (51%)	--
n		--	--	--	19 (6%)
v		--	--	--	3 (1%)
u		--	--	--	1 (0%)
Gender					
Male	631 (76%)	154 (75%)	15 (60%)	198 (78%)	264 (77%)
Female	194 (24%)	50 (25%)	10 (40%)	55 (22%)	79 (23%)
Age					
<30	84 (10%)	24 (12%)	0 (0%)	29 (11%)	31 (9%)
30–40	420 (51%)	103 (51%)	10 (40%)	138 (54%)	169 (49%)
40–50	275 (33%)	66 (33%)	9 (36%)	76 (30%)	124 (36%)
>50	45 (5%)	10 (5%)	6 (24%)	10 (4%)	19 (6%)
Route of Transmission					
Sexual	178 (22%)	39 (19%)	4 (16%)	52 (21%)	83 (24%)
Blood	58 (7%)	35 (17%)	14 (56%)	3 (1%)	6 (2%)
IVDU	566 (69%)	118 (58%)	5 (20%)	191 (75%)	252 (73%)
Unknown	22 (3%)	11 (5%)	2 (8%)	7 (3%)	2 (1%)
Median CD4 count* (range)	126 (31–241)	103 (24–211)	198 (121–338)	150 (42–254)	115.5 (30–237)
Median ALT	38 (27–60)	38.5 (27–67)	30 (24–41)	38 (26–61)	37.5 (27–57)
Median AST	35 (20–58)	36 (20–63)	21 (15–37)	33 (20–50)	34 (20–58)
Median Log10 HCV RNA	6.1 (5.5–6.6)	6.2 (5.6–6.7)	6.1 (5.5–6.6)	5.9 (5.4–6.4)	6.3 (5.7–6.8)

CD4 count expressed as cells/uL (n = 813); ALT = alanine aminotransferase (units/L) (n = 561); AST = aspartate aminotransferase (units/L) (n = 561); HCV RNA expressed as IU/mL (n = 415)

*Obtained at time of enrollment in study

### Rates of natural polymorphisms conferring NS3/4A protease inhibitor resistance

The NS3 region was adequately sequenced for 778 out of 826 patients: genotype 1 (193 of 204), genotype 2 (23 of 25), genotype 3 (237 of 253) and genotype 6 (325 of 344). Naturally occurring RAVs associated with PI resistance were found in all genotypes under study (see Table 2). 81.2% (632/778) of all sequences harbored at least one RAV. One or more RAVs were found in 3.6% (1/28) of genotype 1a, 3.6% (6/165) of genotype 1b, 100% (23/23) of genotype 2a, 100% (125/125) of genotype 3a, 100% (112/112) of genotype 3b, 98.7% (299/303) of genotype 6a, and none (0/22) of genotype 6n/u/v sequences. Variants existing in combination were present in 100% of genotype 3a (V36L + Q80R + D168Q (1); V36L + A156G + D168Q (1); V36L + D168Q (123)) and 3b sequences (V36L + T54S + D168Q (2); V36L + D168Q (110)) and 2.3% of genotype 6a sequences (Q80K + 168E (4); Q80K + I132V (3)). A number of amino acid variants not associated with resistance at positions Q80, I132, and V170 were detected for all genotypes.

**Table 2:** Summary of NS3 protease region resistance-associated variants by genotype.

Residue	V36	Q41	F43	T54	V55	Q80	R109	I132	R155	A156	D168	V170
Genotype												
**1a** (n = 28)	**36A** (1)											170I (28)
**1b** (n = 165)		41H (1)		**54S** (4)		**80R**(1) 80L (1) 80E (3)		132V (79)^+^ 132L (2)			**168E** (1)	170I (75)
**2a** (n = 23)	**36L** (23)					80G (23)		132L (23)				170I (23)
**3a** (n = 125)	**36L** (125)					**80R** (0.5)*		132L (125)		**156G** (1)	**168Q** (125)	170I (120)
**3b** (n = 112)	**36L** (112)			**54S** (2)				132L (112)			**168Q** (112)	170I (112)
**6a** (n = 303)	36I (1)					**80R** (1) **80K** (298)		**132V** (3)			**168E** (4)	170I (303)
**6n** (n = 19)												
**6u** (n = 1)								132L (1)				
**6v** (n = 2)								132L (2)				

*Nucleic acid ambiguity resulting in two possible amino acid conversions, one WT and one mutated, thus characterized as 0.5

^+^Considered significant RAV in genotype 1a replicon only; wild-type in genotype 1b replicon

Variants in bold lettering have been shown to have resistance to NS3 protease inhibitors in vitro and in vivo; only these variants were included in calculation of RAV prevalence for each genotype

Variants without bold lettering are present at residues implicated in resistance, but amino acid change not known to confer resistance

### Positions of NS3/4A resistance-associated variants and correlation with phenotypic resistance data

The V36A variant conferring a 7.4-fold increase in EC_50_ with telaprevir use was detected in only one patient with genotype 1a. V36L, also associated with low-level telaprevir resistance, was present in all genotype 2a, 3a, and 3b sequences. Four patients with genotype 1b and two patients with genotype 3b harbored the T54S variant, which confers low-level resistance to both first generation PIs telaprevir and boceprevir. The Q80K variant with reduced susceptibility to second-generation drug simeprevir was present in 98.4% of genotype 6a sequences. The increase in EC_50_ for Q80K as demonstrated in genotype 1 replicons is 7.8. A much less common variant conferring low-level resistance to simeprevir, Q80R, appeared in genotypes 1b, 3a and 6a. I132V, a low-level RAV that affects telaprevir response in the genotype 1a replicon but not genotype 1b, was observed in 3 patients with genotype 6a.

High-level RAVs other than D168Q were rare, occurring only in 6 (0.8%) patients. Five patients, including one genotype 1b patient and four genotype 6a patients, carried the D168E variant. One genotype 3a patient carried A156G. Both D168E and A156G confer high-level resistance to simeprevir with a respective 38- and 19-fold decreased susceptibility. D168Q confers a fold change in EC_50_ of >700 [25] and is found in all genotype 3 sequences in our cohort. Notably, we did not detect the R155K variant implicated in high-level resistance to all protease inhibitors in any sequence.

### Rates of natural polymorphisms conferring NS5B polymerase inhibitor resistance

The NS5B region was sequenced in 350 of 778 sequences. All sequences were obtained from the Guangdong province of China. Naturally occurring RAVs associated with NS5B resistance were found in several genotypes (see Table 3). 20.6% (72/350) of all sequences harbored at least one RAV. One or more RAVs were found in 92.8% (64/69) of genotype 1b, 5.1% (2/39) of genotype 3a, 4% (1/25) of genotype 3b, 2.5% (5/201) of genotype 6a, and none of genotype 1a/2a/6n sequences. Only one sequence with genotype 3a carried two RAVs.

**Table 3:** Summary of NS5B polymerase region resistance-associated variants by genotype.

Residue	L159	S282	C316	V321
Genotype				
**1a** (n = 10)				
**1b** (n = 69)			**C316N** (64)	
**2a** (n = 4)				
**3a** (n = 39)			**C316N** (1)	V321F (1)
**3b** (n = 25)			C316W (1)	
**6a** (n = 201)			**C316N** (5)	
**6n** (n = 2)				

Variants in bold lettering have been shown to have resistance to NS3 protease inhibitors in vitro and in vivo; only these variants were included in calculation of RAV prevalence for each genotype

Variants without bold lettering are present at residues implicated in resistance, but amino acid change not known to confer resistance

### Positions of NS5B resistance-associated variants and correlation with phenotypic resistance data

Sixty-four patients with genotype 1b, one patient with genotype 3a, and one patient with genotype 6a harbored the C316N variant, which confers low-level resistance to dasabuvir [26] and has been implicated in reduced response rates to sofosbuvir [27]. Two other variants identified—the V321F variant in two patients with genotype 3a and the C316W variant in one patient with genotype 3b –are not associated with phenotypic resistance. No sequence contained at baseline the S282T RAV associated with sofosbuvir resistance in clinical trials [28].

## Discussion

Drug resistance is a major public health threat. Better understanding variants conferring resistance to direct acting antivirals (DAAs) among HCV-infected individuals has important implications for expanding HCV programs worldwide. Our data suggest substantial variation by genotype in the frequency and diversity of natural polymorphisms conferring resistance to NS3/4A protease inhibitors and NS5B polymerase inhibitors in Chinese patients with HIV/HCV co-infection. While baseline resistance analysis on non-genotype 1 sequences is available [29–31], we expand upon this literature with a greater number of individuals with genotypes 3 and 6. Our data also extend the limited literature from China [32]. The high degree of genetic variability amongst HCV genotypes and subtypes has clearly been shown to impact treatment outcomes with DAAs. A deeper understanding of these genetic disparities will assist in achieving superior viral suppression with new and upcoming treatment modalities.

**Fig 2:**
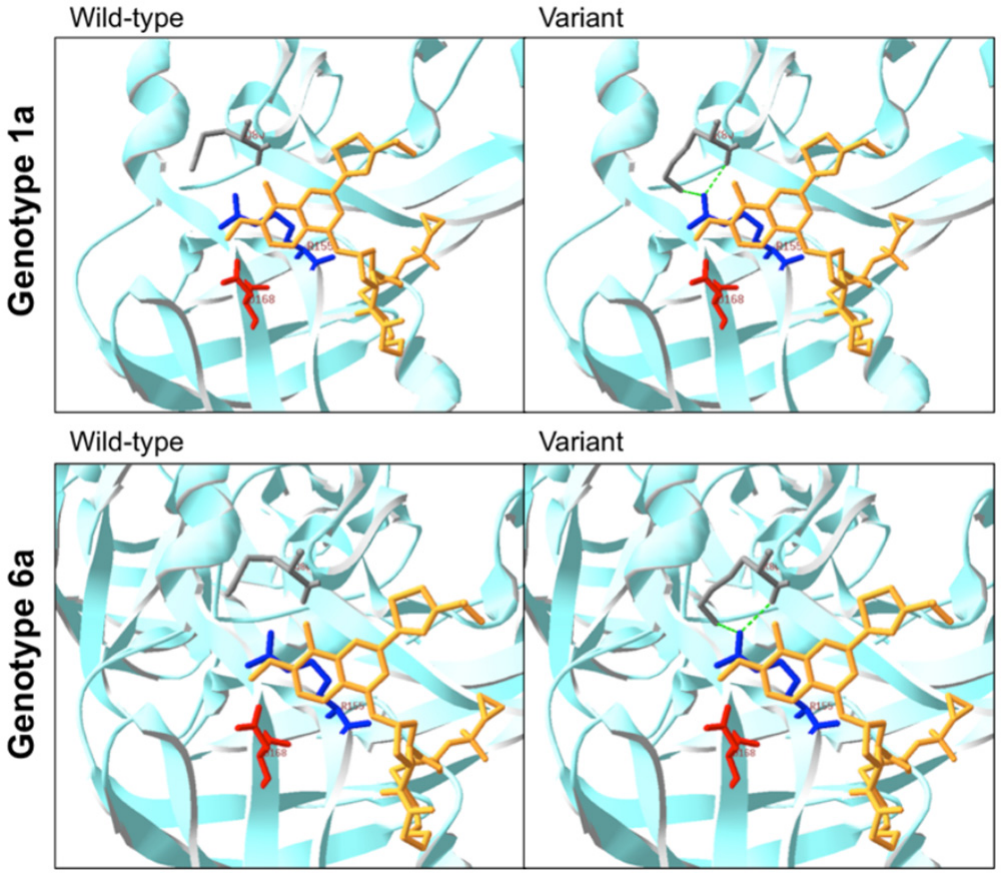
Comparison of crystal structures of NS3 protease with and without the Q80K variant complexing with TMC-435350 (Simeprevir). Similar changes in structure are seen in both genotype 1a and genotype 6a. NS3 protease is shown as light blue ribbon. Yellow structure represents simeprevir (TMC-435350). Residues pictured are as follows: gray (Q80), blue (R155) and red (D168).

We report the presence of the Q80K variant, associated with decreased response to simeprevir in genotype 1a patients, in the majority of individuals with genotype 6a. Endemic to Southeast Asia, genotype 6a is the second most common genotype behind genotype 1b in Southern China [33]. Liu et al. reported similar findings with variant Q80K present in 95% of mono-infected HCV patients in their patients with genotype 6a [32]. The importance of this RAV, seen in 48.1% of genotype 1a patients in North America [34], lies in its influence on clinical response to simeprevir in patients with genotype 1a. Response to simeprevir in patients with the Q80K variant was not statistically significant compared to placebo in the QUEST-1/QUEST-2 trials [35]. A pooled 58.3% of patients with the Q80K variant attained sustained virologic response with simeprevir compared to 83.6% without [35]. The effect of Q80K, however, does not appear to be class-specific given equivalent response to macrocyclic PI faldaprevir [36]. The theory behind decreased efficacy of simeprevir in the presence of Q80K is related to the direct contact between Q80K and R155, which participates in the formation of a salt bridge with residue D168. The disruption of this R155-D168 salt bridge by the Q80K variant leads to a weakened interaction between the protease and the inhibitor and subsequently decreased susceptibility [37]. Our modeling of the Q80K variant in genotype 6a reveals the same structural changes that occur in genotype 1a (Fig 2), which suggests that genotype 6a patients may also have decreased response to simeprevir. However, a phase IIa open-label study on simeprevir monotherapy demonstrated potent antiviral activity against a small cohort of genotype 6 patients, although pretreatment Q80K was present in only one patient [25]. The same study found potent antiviral activity in genotype 4 patients, corroborated by preliminary results from a Phase III trial in genotype 4 patients demonstrating excellent response to simeprevir in combination with peg-interferon and ribavirin, although with higher SVR rates seen in treatment-naïve and relapsed patients than non-responders [38]. These findings lend credence to studying the use of simeprevir in genotype 6 as well. The recent creation of a hepatitis C replicon encoding a genotype-6a NS3 protease should also facilitate phenotypic resistance testing and clarify impact of the Q80K variant [39].

Our data suggest a low frequency and diversity of natural polymorphisms conferring PI resistance in genotype 1a individuals, which contrast with other literature demonstrating up to 29% prevalence (S3 Table). However, our overall RAV rate of 3.6% is consistent with rates reported in a recent review article of 0.1% to 3.1% [40]. Only one clinically significant variant (V36A) was detected in genotype 1a compared to results from resistance studies on clinical trial patients showing a much higher number and variety of RAVs in 1a patients [41–43]. An additional distinction in our genotype 1 population was the absence of the Q80K variant in genotype 1a sequences. The idea of two distinct clades of subtype 1a has been proposed due to the much lower prevalence of the Q80K variant in Europe compared to North America [44]. A recent study from Brazil also did not detect Q80K in a large sample of genotype 1a patients [45], suggesting the potential for additional clades. In general, studies worldwide on PI resistance differ with respect to number and type of variants studied, prior HCV treatment status and presence of HIV co-infection, leading to limited conclusions on regional differences in resistance patterns.

We identified the NS5B polymerase inhibitor variant C316N in the majority of genotype 1b patients at baseline. While NS5B resistance has been rare in clinical trials, the C316N variant has been identified as a baseline polymorphism associated with sofosbuvir resistance in genotype 1b patients (4 of 6 patients also had the L159F variant) [27]. In phase 3 trials with sofosbuvir, genotype 1b patients (82%) also had lower SVR rates than genotype 1a (92%) [46]. A proposed mechanism based on structural modeling is a bulkier polymerase structure involving C316N in 1b patients that may interfere with sofosbuvir binding [27]. Our results are consistent with findings in Chinese genotype 1b HCV mono-infected patients in which the C316N RAV was found in 85.6% (137/160) of sequences[47]. These results taken together suggest DAA regimens without sofosbuvir may be preferable in Chinese genotype 1b patients with and without co-infection. Our study was limited to detecting 4 of 12 known NS5B RAVs. Dedicated study of NS5B polymerase inhibitor resistance in co-infection will be an important area for future research.

Other than the previously described D168Q variant leading to simeprevir non-response in all patients with genotype 3 [25], few high-level RAVs were found (D168E, D168Q and A156G), and all confer resistance to simeprevir only. Few studies on PI resistance have included a substantial number of genotype 3b patients, and interestingly we found the presence of the D168Q variant in all 112 patients. The systematic presence of this variant in genotype 3 confers complete non-response to the macrocyclic protease inhibitor simeprevir, related to structural changes that constrain proper binding [25]. Correspondingly, all genotype 3 patients demonstrate no response to simeprevir use *in vivo* [25]. These findings suggest that primary PI resistance, other than to simeprevir for genotype 3 patients, would be limited in China.

The influence of HIV co-infection on the presence of baseline HCV variants was not addressed in our study but warrants discussion. Rates of RAVs to PIs have been previously established to be largely comparable in HIV/HCV co-infection regardless of HIV treatment status [31, 45, 48]. Furthermore, co-infected patients exposed to an HIV regimen including a protease inhibitor also did not exhibit higher RAV rates [49–51]. Of note is a small study on co-infected patients reporting sites of primary resistance to first-generation HCV PIs in two patients on PI-containing HIV therapy [52], raising the concern of selective pressure. While larger studies are merited, these findings suggest that from a resistance standpoint HIV co-infection may not be an additional hindrance to HCV treatment options. A further limitation in our study is the application of resistance data from genotype 1 to other genotypes, and accurate conclusions on phenotypic or clinical resistance cannot be made. The ongoing creation of non-genotype 1 replicons and ensuing results from phenotypic experiments will inform future resistance studies. Lastly, our use of population sequencing does not provide as complete a picture of the HCV viral quasispecies population as can now be characterized using next generation sequencing. Due to the limitations of our sequencing method, it is possible that sequences harboring high-level RAVs in our patients escaped detection and have the potential to become the dominant population under selective pressure from treatment. As such, we may be underestimating the true prevalence of high-level RAVs. The rapidly increasing application of next generation sequencing will be paramount to the understanding of HCV resistant variant dynamics prior to, during and after treatment with DAAs and an area for future study.

In summary, we report an overall 72.8% prevalence of preexisting resistance-associated variants to NS3/4A protease inhibitors and 20.6% to NS5B polymerase inhibitors in a large cohort of treatment-naïve HIV/HCV co-infected patients from China. Few sequences (0.8%) harbored high-level RAVs capable of causing primary DAA resistance. The Q80K variant associated with decreased efficacy of simeprevir was nearly universally present in patients with genotype 6a. Similarly nearly all genotype 1b patients harbored the C316N variant associated with decreased response to sofosbuvir. Clinical trials with these DAAs will be necessary prior to implementation in China. Further phenotypic studies and clinical research on a broad spectrum of genotypes as well as patients co-infected with HIV are still needed.

## Supporting Information

S1 Table Reference sequences used for HCV genotyping and subtyping. (DOCX)

S2 Table HCV primers for NS3/4A region by genotype. (DOCX)

S3 Table Comparison of previously published literature on prevalence of NS3 protease inhibitors baseline RAVs in HCV mono-infected and HIV/HCV co-infected populations. (DOCX)
