# Supplementary material for: Natural Polymorphisms Conferring Resistance to HCV Protease and Polymerase Inhibitors in Treatment-Naïve HIV/HCV Co-Infected Patients in China
Source: PLoS One. 2016 Jun 24;11(6):e0157438. doi: 10.1371/journal.pone.0157438 (PMC4920402; doi:10.1371/journal.pone.0157438)
Supplement: S1 Table — (DOCX) [file pone.0157438.s001.docx]

**S1 Table - Reference sequences used for HCV genotyping and subtyping**

Genotype Reference sequence number

1a AB520610 EU781769 EU781824 FJ205868 M62321 M67463

1b D10934 L02836 M58335

1c AY051292 AY651061 D14853

2a AF238482 AF238483 D00944

2b AB030907 AF238486 D10988

2c D50409

3a AF046866 D17763 D28917

3b D49374

4a Y11604

5a Y13184

6a AY859526 DQ480515 DQ480522 Y12083

6b D84262

6n DQ278894 HQ229167(Core)

6u EU408331

6v EU158186 EU798760 EU798761 FJ435090
