# Supplementary material for: Natural Polymorphisms Conferring Resistance to HCV Protease and Polymerase Inhibitors in Treatment-Naïve HIV/HCV Co-Infected Patients in China
Source: PLoS One. 2016 Jun 24;11(6):e0157438. doi: 10.1371/journal.pone.0157438 (PMC4920402; doi:10.1371/journal.pone.0157438)
Supplement: S2 Table — (DOCX) [file pone.0157438.s002.docx]

**S2 Table - HCV primers for NS3/4A region by genotype.**

| **Genotype 1a** | | |
| --- | --- | --- |
| Outside Primers(5’---3’) | Sense | Position |
| CCRRATGGAGACCAAGMTCATYACGT | + | 3275---3300 |
| TCNGTDGARTGGCACTCRTCACA | - | 4309---4287 |
| Inside Primers(5’---3’) |  |  |
| GCRTGYGGBGACATCATYAACGG | + | 3319---3341 |
| CAVCCRCCRTCRGCAAGGAACTTG | - | 4258---4235 |
| **Genotype 1b** | | |
| Outside Primers(5’---3’) | Sense | Position |
| GAYATGGAGAYYAAGRTCATYACCTGG | + | 3278---3304 |
| TCCAGCCGTCTCCGCTTGG | - | 4366---4348 |
| Inside Primers(5’---3’) |  |  |
| GCRGCRTGYGGGGACATCAT | + | 3317---3336 |
| CCARGACYGTGCCRATGCCCA | - | 4346---4326 |
| **Genotype 2a** | | |
| Outside Primers(5’---3’) | Sense | Position |
| CTCACMCCTATGTCGGAYTGGGC | + | 3230---3252 |
| CCATCGGCGAGRAATTTGCCATAYGTGGA | - | 4282---4254 |
| Inside Primers(5’---3’) |  |  |
| ATGGAGAAGAAGGTCATCGTCTGGGG | + | 3308---3333 |
| GTCCTAATGTTGGGATTGATGCCATGTGC | - | 4213---4185 |
| **Genotype 3a** | | |
| Outside Primers(5’---3’) | Sense | Position |
| GAYCACCTAGCRCCAATGCAACA | + | 3217---3239 |
| TAGGTGGARTAGGTCAGTTTRGCACCAGTTG | - | 4253---4223 |
| Inside Primers(5’---3’) |  |  |
| GCCACTGAACCTGTAATATTTAGTCCCATGG | + | 3274---3304 |
| GTGCGGTTCCCAGTGCGGA | - | 4217---4199 |
| **Genotype 3b** | | |
| Outside Primers(5’---3’) | Sense | Position |
| GGGCYGCYGMHGGYCTCAAAG | + | 3247---3267 |
| CGTCARCCTYACCCCAGCTGTCTC | - | 4443---4380 |
| Inside Primers(5’---3’) |  |  |
| CCYATGGARATYAAGGTYATYACYTGGGG | + | 3303---3331 |
| GGGTCAATCCCATAGGCTTTCGACATG | - | 4198---4172 |
| **Genotype 6a** | | |
| Outside Primers(5’---3’) | Sense | Position |
| TGRCTGGGCTAGCGACGGACT | + | 3207---3227 |
| TGGCACTCRTCRCAGATGATGATGTC | - | 4292---4267 |
| Inside Primers(5’---3’) |  |  |
| ATGCRGCGYGGRGGTTGGAAG | + | 3385---3415 |
| GYCTCATRTAWGAGCCRAAGCTAAGCGT | - | 4143---4116 |
| **Genotype 6n, 6u, 6v** | | |
| Outside Primers(5’---3’) | Sense | Position |
| GAYMTVGCYGTHGCBGTYGARCC | + | 3269---3291 |
| GGRTCHGTDGARTGRCAYTCGTCACA | - | 4342---4317 |
| Inside Primers(5’---3’) |  |  |
| CHATGGAGAARAARRTYATCACCTGGGG | + | 3305---3333 |
| CCBCCDGARCAWCCMCCRTC | - | 4296---4277 |
