# Supplementary material for: Natural Polymorphisms Conferring Resistance to HCV Protease and Polymerase Inhibitors in Treatment-Naïve HIV/HCV Co-Infected Patients in China
Source: PLoS One. 2016 Jun 24;11(6):e0157438. doi: 10.1371/journal.pone.0157438 (PMC4920402; doi:10.1371/journal.pone.0157438)
Supplement: S3 Table — (DOCX) [file pone.0157438.s003.docx]

**S3 Table - Comparison of previously published literature on prevalence of NS3 protease inhibitors baseline RAVs in HCV mono-infected and HIV/HCV co-infected populations.**

|  | **Reference (year)** | **Sample size** | **HIV** | **Prior treatment status** | **Study site** | **Geno-type** | **RAV prevalence*** | **RAVs identified (# of patients if available)** |
| --- | --- | --- | --- | --- | --- | --- | --- | --- |
| 1 | Bartels et al. (2008) | 570 | No | Naïve to PI | Clinical trial (international) | 1a | 2.8% | V36M (5), R109K (1), R155K (4), V170A (1) |
|  |  |  |  |  |  | 1b | 0% |  |
| 2 | Bartels et al. (2013) | 3,447 | No | Naïve to PI | Clinical trial (international) | 1a | 0-3% | V36I/L/M, F43Y, T54A/S, V55A/I, Q80K/R, V107I, I132V, R155K, D168E, I170T |
|  |  |  |  |  |  | 1b | 0-3% | V36I/L/M, T54S, V55A, Q80K/R, V107I, V158I, D168E, I170A/T, M175L |
| 3 | Kuntzen et al. (2008) | 507 | No | Naïve | USA Switzerland Germany | 1a | 8.6% | V36L (6), V36M (2), T54S (7), R155K (3), D168E (1) |
|  |  |  |  |  |  |  |  |  |
|  |  |  |  |  |  | 1b | 1.4% | T54S (2) |
| 4 | Gaudieri et al. (2009) | 406 | Yes^+^ | HCV-Naïve HIV-Unknown | Australia Switzerland United Kingdom | 1a | 16.2% | V36L, Q41H, T54S, R109T, I153L, R155T, D168E, I170V, E176K |
|  |  |  |  |  |  | 1b | 10.8% | I153V, V160I |
|  |  |  |  |  |  | 3a | 18.3% | T54S, Q168R, I170V, S176N, S176G |
| 5 | Andanov et al. (2013) | 85 | No | Naïve to PI | Canada | 1a | 20% | V36L, V36M, T54S, V55A, V55I, A156G |
| 6 | Trimoulet et al. (2011) | 120 | Yes | HCV-Naïve to PI HIV-ART | France | 1 | 6.9% | V36L (1), V36M (2), T54S (2), R155K (1) |
|  |  |  |  |  |  | 4 | 100% | V36L (44) |
| 7 | Vallet et al. (2011) | 233 | Yes^+^ | HCV-Naïve to PI HIV-Unknown | France | 1a | 5% | T54S, V36L, Q80K, D168E, V170I |
|  |  |  |  |  |  | 1b |  | V36L, T54S, Q80K/R/L, V170I/T |
|  |  |  |  |  |  | 2 | 100% | V36L, Q80G, V158V/I, V170I |
|  |  |  |  |  |  | 3 | 100% | V36L (all), D168Q (all), V170I |
|  |  |  |  |  |  | 4 | 100% | T54S, V36L (all), V170I |
|  |  |  |  |  |  | 5 | 100% | DV36L, Q41H, Q80K, V158L/M, D168E, V170I |
| 8 | Morsica et al. (2009) | 37 | Yes | HCV-Naive HIV-ART | Italy | 1a | 17.9% | R155K (2), A156T (1), V170F (2), V170E/T/Y (1) |
|  |  |  |  |  |  | 1b | 12.5% | A156T (1) |
|  |  |  |  |  |  | 1c | 0% |  |
| 9 | Paolucci et al. (2012) | 156 | Yes^+^ | HCV-Naïve to PI HIV-ART | Italy | 1a | 29% | V36L (2), T54S (2), V55A/I (2), Q80K/L (3 (2) |
|  |  |  |  |  |  | 1b | 10% | V55F (1), Q80L/N (2), M175L (1) |
|  |  |  |  |  |  | 2 | 3% | V158M (1) |
|  |  |  |  |  |  | 3 | 100% | D168Q (33) |
|  |  |  |  |  |  | 4 | 13% | D168E (3) |
| 10 | Vicenti et al. (2012) | 109 | Yes^+^ | HCV-Naïve HIV-Unknown | Italy | 1a | 25.4% | V36L (4), T54S (2), V55A (1), Q80K (11) |
|  |  |  |  |  |  | 1b | 9.5% | V36L (2), T54S (1), V55A (1) |
| 11 | Trevino et al. (2011) | 55 | Yes | HCV-Naive HIV-Naïve | Spain | 1a | 62% | Q80K (8), V55A (2) |
|  |  |  |  |  |  | 1b | 30% | Q80K (1) |
| 12 | Palanisamy et al. (2013) | 126 | No | Naïve to PI | Sweden | 1a | 28% | V36L (2), T54A (1), T54S (4), V55A (4), V55I (3), Q80K (3), Q80R (2) |
|  |  |  |  |  |  | 1b | 0% |  |
|  |  |  |  |  |  | 2b | 9.1% | T54S (1) |
|  |  |  |  |  |  |  | 100% | V36L (11), Q80G (11), S122T (11) |
|  |  |  |  |  |  | 3a | 100% | V36L (30) |
| 13 | Zabek et al. (2013) | 85 | No | Naïve | Poland | 1b | 14.1% | T54S (2), A87T (2), R117H (2), V55A (1), D168E (5) |
| 14 | Peres-da-Silva et al. (2010) | 114 | No | Naïve | Brazil | 1a | 4.1% | T54S (2), V170I (46) |
|  |  |  |  |  |  | 1b | 5.6% | V36L (3), V170I (19) |
|  |  |  |  |  |  | 3a | 100% | V36L (13), D168Q (13), V170I (13) |
| 15 | De Carvalho et al. (2014) | 171 | No | Unknown | Brazil | 1a | 7.4% | V36L, T54S, Q80K, R155K |
|  |  |  |  |  |  | 1b | 5.1% | V36L, Q41R, T54S, D168S |
| 16 | Lisboa-Neto et al. (2014) | 247 | Yes^+^ | HCV-Naïve to PI HIV-ART | Brazil | 1 | 21.9% | M175L (3), S122G (28), S122N (3), S122T (3), T54S (5), V55A (3), V55I (4), Q80L (4), Q80R (2), V36L (1), V55A (4) |
| 17 | Nishiya et al. (2014) | 138 | No | Naïve | Brazil | 1a | 20% | V36L (2), V55A (3), Q80L (1), R155K (5) |
|  |  |  |  |  |  | 1b | 8% | T54S (2), V55A (1), R117H (2), D158G (1) |
| 18 | Shindo et al. (2011) | 261 | No | Naïve | Japan | 1b | 13.4% | T54S (14), Q80K (1), I153V (22), D168E (4) |
| 19 | Suzuki et al. (2012) | 362 | No | Naïve to PI | Japan | 1b | 4.9% | V36A (1), T54S (10), Q80R (2), D168E (2) |
| 20 | Larousse et al. (2014) | 131 | No | Naïve | Tunisia | 1a | 14.2% | R155K (1) |
|  |  |  |  |  |  | 1b | 8.9% | T54S (2), V55A (2), Q80K (4), A156V (1) |
| 21 | Liu et al. (2014) | 162 | No | Naïve | China | 1b | 38.30% | T54S (4), Q80L (2), A156S (11), D168Y (1), V170I (10) |
|  |  |  |  |  |  | 2a | 100.00% | V36L (14), Q80G (14), A156S (9), V170I (14) |
|  |  |  |  |  |  | 6a | 100.00% | V36L (4), Q80K (84), D168E (2), V170I (87) |

*Percentages obtained directly from text or calculated from tables, with an attempt to classify by genotype and subtype when data available

^+^Both mono-infected and co-infected cohorts studied

Search performed using PubMed in December 2014 using search terms “hepatitis C virus”, “protease inhibitor”, and “resistance”.
